# Effects of Cumulative Low-Density Lipoprotein Cholesterol Exposure on Endothelial Function in Subjects Receiving Lipid-Lowering Drugs

**DOI:** 10.1016/j.jacasi.2025.02.015

**Published:** 2025-04-15

**Authors:** Takayuki Yamaji, Farina Mohamad Yusoff, Shinji Kishimoto, Masato Kajikawa, Takahiro Harada, Aya Mizobuchi, Shunsuke Tanigawa, Tatsuya Maruhashi, Ayumu Nakashima, Yukihito Higashi

**Affiliations:** a Center for Radiation Disaster Medical Science, Research Institute for Radiation Biology and Medicine, Hiroshima University, Hiroshima, Japan; b Division of Radiation Medical Science, Department of Regenerative Medicine, Research Institute for Radiation Biology and Medicine, Hiroshima University, Hiroshima, Japan; c Division of Regeneration and Medicine, Medical Center for Translational and Clinical Research, Hiroshima University Hospital, Hiroshima, Japan; d Department of Nephrology, Graduate School of Medicine, University of Yamanashi, Yamanashi, Japan

**Keywords:** cumulative low-density lipoprotein cholesterol, endothelial function, flow-mediated vasodilation, lipid lowering drug

Endothelial dysfunction occurs in the early stage of atherosclerosis progression and leads to cardiovascular events.^1^ Measurement of flow-mediated vasodilation (FMD) in the brachial artery is one of the most common tools for assessing endothelial function noninvasively.^1^ Recently, cumulative low-density lipoprotein cholesterol (LDL-C) exposure has been reported to be a stronger predictor than LDL-C level for cardiovascular events.^2^ On the other hand, information on the relationship between endothelial function and cumulative LDL-C exposure has been limited. Therefore, in a previous study, we assessed the correlation between cumulative LDL-C exposure, calculated by age × LDL-C, and the progression of atherosclerosis in subjects who were not receiving lipid-lowering drugs, and we showed that endothelial dysfunction began at a cumulative LDL-C exposure of 4,000 mg.y/dL.^3^ However, there is still no information on the relationship between cumulative LDL-C exposure and FMD in patients receiving lipid-lowering drugs. Furthermore, there is no information on the relationship between lowering LDL-C exposure and improvement of FMD. Therefore, in the present study, we evaluated the long-term effects of cumulative LDL-C exposure on endothelial function in patients receiving lipid-lowering drugs.

This study was a retrospective cohort study of subjects in FMD-J study B, which was a prospective multicenter observational cohort study in Japan to examine the usefulness of FMD assessment for management of patients with a 3-year follow-up period.^4^ A total number of 203 subjects who were taking lipid-lowering drugs and who had no history of cardiovascular disease were included. The ethical committees of the participating institutions approved the study protocol. The study was executed in accordance with the Good Clinical Practice guidelines. All subjects gave written informed consent for participation in the study. The protocol was registered in the University Hospital Medical Information Network Clinical Trials Registry (UMIN000012951). A high-resolution linear artery transducer was coupled to computer-assisted analysis software (UNEXEF18G, UNEX Co) that used an automated edge detection system for measurement of the brachial artery diameter. Percentage of FMD ([peak diameter − baseline diameter]/baseline diameter) was used for analysis. FMD was measured at baseline and 3 years later. Improvement of FMD was defined as (FMD at 3 years − FMD at baseline) >0%. LDL-C was measured at baseline and at 18 months and 3 years later. The time-weighted cumulative LDL-C was calculated as: ([LDL-C at baseline + LDL-C at 1.5 years]/2 × 1.5 + [LDL-C at 1.5 years + LDL-C at 3 years]/2 × 1.5)/3. Time in target range was calculated by the time below the LDL-C target level for 3 years using the modified method of Wang et al.^5^ To determine the target levels of LDL-C, we set 4 different cutoff values (70, 89.2, 89.5, and 100 mg/dL) and assessed the association between improvement of FMD in each group. The target range of 89.2 mg/dL (AUCOC: 0.60, sensitivity: 0.39, and specificity: 0.80) and 89.5 mg/dL (AUCOC: 0.59, sensitivity: 0.39, and specificity: 0.77) were determined on the basis of the cutoff value of time-weighted cumulative LDL-C and mean LDL-C 3 measurements derived from receiver-operator characteristic curves for predicting an improvement in FMD. Time in target range below 89.5 mg/dL was determined according to the highest Youden index from the ROC curves for predicting improvement of FMD (AUCOC: 0.59, sensitivity: 0.42, and specificity: 0.77).

All reported *P* values were 2-sided, and a *P* value <0.05 was considered statistically significant. A mixed effects logistic regression multivariate logistic regression analysis was performed to identify independent variables associated with improvement of FMD. Age, gender, body mass index, high-sensitivity C-reactive protein, presence of hypertension, diabetes mellitus, current smoking, and exercise habit were entered into the mixed effects multivariate logistic regression analysis, in which the study center variability was treated as random effects. The data were processed using JMP version 16 (SAS Institute).

Among 203 subjects, mean age was 64 years; the proportion of men was 48.8% (99 of the 203); and the prevalence of hypertension was 97.5% (198 of the 203), diabetes mellitus was 26.9% (54 of the 203), and current smokers was 10% (20 of the 200). The median FMD value was 4.9% (Q1, Q3: 2.7%, 6.5%). After adjustment for confounding factors, the odds of having improvement of FMD were significantly higher in the sum of 3 measurements of LDL-C levels of <268.5 mg/dL (mean LDL-C: 89.5 mg/dL) group than in the ≥268.5 mg/dL (mean LDL-C: 89.5 mg/dL) group (OR: 2.12; 95% CI: 1.09-4.12; *P =* 0.028), in the time-weighted cumulative LDL-C ≥ 89.2 mg/dL group than in time-weighted LDL-C <89.2 mg/dL group (OR: 3.01; 95% CI: 1.47-6.19; *P* = 0.003, and in the time in target range below 89.5 mg/dL ≥ 1.5 years group than in the below 89.5 mg/dL <1.5 years group (OR: 3.13; 95% CI: 1.56-6.25; *P* = 0.002) (Figure 1A). For every 1-year increase in time in target range below 70, 89.2, and 89.5 mg/dL, the odds of having an improvement in FMD were significant even after adjustment for confounding factors (OR: 1.97; 95% CI: 1.11-3.52; *P =* 0.022; OR: 1.37; 95% CI: 1.10-1.76; *P =* 0.012; and OR: 1.39; 95% CI: 1.08-1.79; *P =* 0.012, respectively) (Figure 1B).

**Figure 1:**
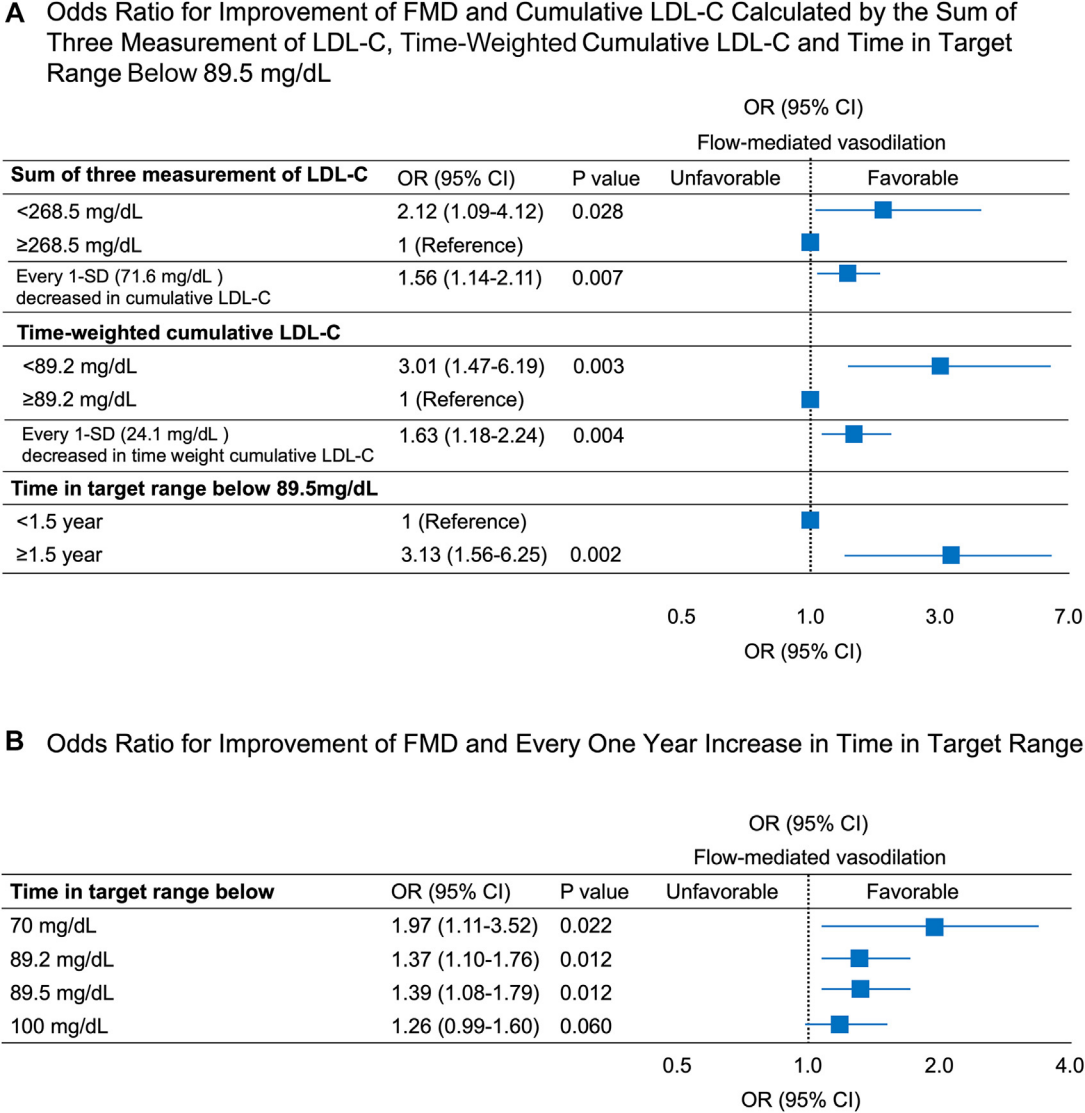
OR for Improvement of FMD and Cumulative LDL-C Odds ratios and 95% CIs for improvement of flow-mediated vasodilation (FMD) for cumulative low-density lipoprotein cholesterol (LDL-C), for time-weighted cumulative LDL-C, and for time in target range below 89.5 mg/dL (A), as well as for every 1 year increased in time in target range (B). The association between cumulative LDL-C exposure and improvement of FMD using a mixed effects logistic regression model in which the study center variability was treated as random effects. Cumulative LDL-C exposure is a strong predictor for improvement of endothelial dysfunction.

The strength of this study was assessment of the optimal target level of cumulative LDL-C for improvement of FMD in patients who are receiving lipid-lowering drugs and those without cardiovascular disease. Cumulative LDL-C exposure calculated by the sum of 3 measurements of LDL-C levels, time-weighted cumulative LDL-C, and time in target range below 89.5 mg/dL were significantly correlated with improvement of FMD. There has been no study showing the optimal target level of LDL-C for improvement of FMD in patients who are receiving lipid-lowering drugs. Thus, we assessed what level of cumulative LDL-C exposure has a beneficial effect on endothelial function. Maintenance of mean LDL-C of <89.5 mg/dL led to an improvement of FMD. These findings suggest that cumulative LDL-C exposure is a strong predictor for improvement of endothelial dysfunction.

However, this study has several limitations. Although this study was conducted in multiple centers, it was not a randomized control study. Second, we did not have information on LDL-C levels before medical therapy and doses of lipid-lowering drugs. Measurement of LDL-C levels before medical therapy would enable more specific conclusions to be drawn. Further studies are needed to assess the association of cumulative LDL-C exposure with endothelial function in a large clinical trial. Measurement of FMD is not routinely available in clinics, and FMD has a large imprecision of measurement. In this study, the FMD measurement device used was simple and highly reproducible, but it still requires some level of examiner skill. In the future, we hope that more user-friendly and reproducible devices that do not require special skills will be developed, allowing FMD measurement to become a routine test even in clinics. Therefore, the practical implications of this study may still be limited at present. This study did not examine whether knowledge of FMD could be used to reduce the burden of LDL-C exposure.

In conclusion, cumulative LDL-C exposure calculated by the sum of 3 measurements of LDL-C levels, time-weighted cumulative LDL-C, and time in target range are associated with changes in FMD, and maintenance of mean LDL-C of <89.5 mg/dL is associated with improvement of FMD.

## Funding Support and Author Disclosures

This work was supported by a Grant-in-Aid for Scientific Research from the Ministry of Education, Science and Culture of Japan (18590815 and 21590898 to Dr Higashi). The authors have reported that they have no relationships relevant to the contents of this paper to disclose.
